# Protective Effects of Thymoquinon on Pulmonary Disorders in Experimental Studies

**Published:** 2018-10

**Authors:** Mohammad-Foad Noorbakhsh, Hanieh Shaterzadeh-Yazdi, Farzad Hayati, Saeed Samarghandian, Tahereh Farkhondeh

**Affiliations:** 1 Division of Pharmacology and Toxicology, Department of Basic Sciences, School of Veterinary Medicine, Shiraz University, Shiraz, Iran,; 2 Research Center of Pardis Hospital, Mashhad, Iran,; 3 Noncommunicable Disease Research Center, Neyshabur University of Medical Sciences, Neyshabur, Iran,; 4 Department of Basic Medical Sciences, Neyshabur University of Medical Sciences, Neyshabur, Iran,; 5 Cardiovascular Diseases Research Center, Birjand University of Medical Sciences, Birjand, Iran.

**Keywords:** Thymoquinone, Lung inflammation, Asthma, Lung fibrosis, Lung cancer

## Abstract

Lung as vital organ is exposed to many injurious agents that can cause inflammation and oxidative stress, which are potential causes in the pathogenesis of lung diseases. *Nigella sativa,* usually introduced as black seed, has been considered for treatment of various diseases and is one the most widely investigated herbs. Thymoquinone (TQ) is the major component of the volatile oil of black seed (54%) which has been indicated to anti-oxidant, anti-inflammatory and anti-neoplastic properties. There is interesting to study on TQ effect as a therapeutic agent for various diseases in both *in vivo* and *in vitro* conditions. In this comprehensive review, we summarized the recent studies related to the effectiveness of TQ on lung disorders such as inflammatory lung diseases, lung fibrosis, asthma and lung cancer. It is concluded that TQ with anti-inflammatory, anti- oxidant, anti-asthmatic and anti- tumor activity can provide therapeutic effects against lung disorders. However, more investigation is needed to produce TQ as a pharmaceutical preparation for human studies.

## INTRODUCTION

Nigella sativa (*N. sativa)* is an annual herb that grows in the Pakistan, India and countries surrounding the Mediterranean Sea (1). The seed is named black cumin or black seed in English. Black seed has been applied for heart support, liver health, respiratory health, digestive issues and also to treat rheumatism, bronchitis, asthma and other inflammatory disorders (2). Black seed consists of over 100 valuable ingredients. It is a rich source of proteins, essential fatty acids, carbohydrates, minerals and vitamins (3). The active components of black seed are Thymoquinone (TQ), dithymoquinone, thymol, thymohydroquinone, nigellone, and fixed oils. TQ and nigellone are two of the most volatile oils which are found in black seed (4, 5). Oxidative stress has an important role in the pathogenesis and progression of pulmonary disorders. Pulmonary diseases such as Chronic Obstructive Pulmonary Disease (COPD), asthma, lung fibrosis and lung cancer, are known as causes of morbidity and death all over the world and could occur following abnormal inflammatory process (6, 7). It has been found that TQ has beneficial protective effects against various diseases through anti-inflammatory, anti-oxidant and anti- apoptotic activities. The present review provides the recent studies from 2005 to 2017 regarding the protective effects of TQ in the management of pulmonary diseases.

## METHODS

Databases such as PubMed, Science Direct, EMBASE, Scopus and Google Scholar were searched for the terms of thymoquinone, lung inflammation, asthma, pulmonary fibrosis, lung cancer between the years 2005 and 2017 to prepare this review. Searching was done on articles in English language.

### Effect of TQ on lung inflammation and injury

Recently, the incidence of lung disorders is considered as important causes of morbidity and mortality all around the world. Most of the lung diseases such as COPD and asthma are described as inflammatory disorders (8). Lung inflammation can occur following challenge to pathogens, toxins, stimuli, allergens and pollutants. There are two types of inflammation including acute (acute pneumonia) and chronic (COPD and asthma). The induction of inflammatory responses in the lung contributes to severe lung disease and causes a serious *threat* to human health. (9–12). TQ, the main active component of the volatile oil of *N. sativa* seeds, has been shown to have anti-inflammatory effects (13, 14).

In this regard, Isik et al. studied the effect of TQ (6 mg/kg, intraperitoneally-i.p) on Acute Lung Injury (ALI) and Acute Respiratory Distress Syndrome (ARDS) induced by human gastric juice in 40 rats. Blood gas analysis and compliance measurement were done. At the end of the third hour, rats were sacrificed and their lungs were excised for histopathological examination. The results indicated that static compliance value was higher in groups treated with TQ alone or in combination with methylprednisolone (10 *mg/kg*, i.p). Also, histopathological study showed that both of them had protective effects on lung against the harmful effects of gastric juice (15). Another study investigated the protective effects of TQ (3 *mg/kg*, i.p, for the 5 days) against Ovalbumin (OVA) which induced airway inflammation in 48 male and female mice. Induction of allergic airway inflammation was performed by intraperitoneal sensitization and airway challenge through nasal inhalation. The results showed that pretreatment with TQ significantly decreased Th2 cytokines, lung eosinophilic inflammation and goblet cell hyperplasia. It also inhibited cyclo-oxygenase-2 (COX-2) protein expression and Prostaglandin D2 (PGD2) generation (16). Keyhanmanesh et al. investigated the prophylactic properties of TQ on the serum levels of IFN-γ and IL-4 and lung histopathology in the 32 OVA-sensitized guinea pigs. Oral administration of TQ (20 and 40 *μM*), ameliorated the pathological changes of lung and the serum levels of IFN-γ and IL-4 in the sensitized animals (17).

Another study indicated the preventive effects of TQ [6 *mg/kg*, i.p, 24 and 1 hour before diesel exhaust particles (30 *μg*) injection] against Diesel Exhaust Particles (DEP) induced cardiopulmonary in mice. For the in vitro experiment, untreated blood was incubated for 3 *min* with various concentrations of TQ (0.01–0.1 *mg/ml*) before adding DEP (1 *μg/ml*) to the blood. Platelet aggregation described above was assessed. The results indicated that pre-treatment with TQ ameliorated Systolic Blood Pressure (SBP), leukocytosis, the serum levels of IL-6 and Superoxide Dismutase (SOD) activity. TQ also retained platelets from a decrease in number, but not platelet aggregation (18).

Another study was done to assess the effect of TQ on lung damages induced by chronic toluene challenge in a rat model that were allotted into one of three experimental groups: control, toluene-treated and toluene-treated with TQ; each group contained 10 animals. The study indicated that TQ (50 *mg/kg*, once a day orally for 12 weeks starting just after toluene exposure) decreased peribronchial inflammation, alveolar edema, alveolar septal infiltration, alveolar exudate, interstitial fibrosis and necrosis following chronic toluene exposure. In addition, the results demonstrated a marked decrease in the action of in situ apoptosis recognition by terminal dUTP nick end-labeling (TUNEL), the inducible Nitric Oxide Synthase (iNOS) and an increase of lung tissue surfactant protein D expression (19).

Suddek et al. investigated the effects of TQ as an antioxidant and anti-inflammatory component against lung oxidative injury induced by Cyclophosphamide (CP) in 32 rats. Administration of TQ (100 *mg/kg/day*, orally) before and after CP (150 *mg/kg*, i.p) injection, significantly decreased the changes in lung and serum parameters via modulating inflammatory responses and oxidative stress. Additionally, TQ decreased secretion of serum Tumor Necrosis Factor (TNF-α) as pro-inflammatory cytokine. Furthermore, TQ reduced lung histopathological alternations induced by CP. This study claimed that TQ has a protective effect against lung injury induced by CP (20).

Aydin and colleagues studied the effect of TQ on heart, kidney and lung tissues in abdominal aorta ischemiareperfusion injury. Thirty rats were divided into three groups as sham (n=10), control (n=10) and TQ treatment group (n=10). Control and TQ-treatment groups underwent abdominal aorta ischemia for 45 *min* followed by a 120-*min* period of reperfusion Pretreatment with TQ (20 *mg/kg*, i.p) before reperfusion, reduced oxidative stress and histopathological damages induced by acute abdominal aorta ischemia-reperfusion (21). Hyperbaric Oxygen (HBO _2 _) therapy increased Reactive Oxygen Species (ROS) production that results in cellular injury. 30 female rats were randomly assigned to one of the three groups (n = 10 per group). Group 1 represented the control group (no treatment). Group 2 was exposed to 100% oxygen at 2.5 ATA for two sessions of two hours’ duration each day for five days. Group 3 was treated identically to group 2 and was also given TQ once daily at 50 *mg/kg/day* by oral gavage for five days, after first session of HBO _2_ . It was indicated that TQ as an antimicrobial and anti-inflammatory agent stopped the free radical generation rat model. Treatment with TQ significantly reduced lipid hydroperoxide (*LOOH)* and total thiol (*SH) levels*. The study suggested that TQ may be effective as a therapeutic agent during HBO _2_ therapy by ameliorating oxidative stress (22).

Ozer et al. investigated the effect of TQ against oxidative and inflammatory damages and Mesenteric Artery Blood Flow (MABF) in a rat sepsis model induced by Cecal Ligation and Puncture (CLP). Rats were divided into the following four groups: Sham, CLP, Sham plus TQ and CLP plus TQ. TQ (1 *mg/kg*) or vehicle (dimethyl sulfoxide, 1 *mL/kg/day*) was i.p injected for 3 days. On the 4th day Sham or CLP operation was applied. 20 hr after the operations, MABF and contractile responses of isolated aortic rings to phenylephrine were measured. Tissue samples were obtained for histopathological and biochemical examinations. Also, survival rates were recorded throughout 96 hr. TQ improved mesenteric hypo-perfusion and decreased CLP induced aortic impairment. Also, TQ inhibited CLP induced elevation of serum Alanine aminotransferase (ALT), Aspartate aminotransferase (AST), Lactate Dehydrogenase (LDH), Creatinine (Cr), Blood Urea Nitrogen (BUN), TNF-α, IL-1 β and IL-6 (as inflammatory cytokines). Furthermore, TQ ameliorated the liver, lung, spleen and kidney levels of GSH and MDA in rat (23).

### Effects of TQ on mucociliary clearance

Airway Surface Liquid (ASL) and cilia, two main parts of the mucociliary system, eliminate particles from the respiratory system. Therefore, mucociliary clearance protects the lungs against the injurious effects of aspirated particles (24–28). Wienkötter et al. investigated the antispasmodic property of nigellone and TQ on trachea and mucociliary clearance in mice. The results showed that both nigellon and TQ had a dose-dependent preventive property on trachea exposed to Ba ^2+^. High TQ concentration (153.0 vs. 505.0 sec /12 *mm* distance) and nigellone also prevented tracheal contraction induced by Leukotriene-d. In addition, the rate of mucociliary transport was slightly improved after TQ. However, the study did not confirm the antispasmodic effect of TQ (29). Another study assessed the effect of TQ and montelukast (as a conventional drug for treating of asthma and allergic rhinitis) on human Cilia Beat Frequency (CBF) in well-differentiated human sinonasal epithelial cells. Well-differentiated human sinonasal epithelial cultures, grown at an air-liquid interface were treated with varying concentrations of TQ and montelukast. Changes in CBF were determined using the Sissons-Ammons Video Analysis system. TQ (50 and 100 μM) improved CBF in dose dependent manner. Also, montelukast had similar effect on CBF. The findings showed that TQ and montelukast improved respiratory disorders by ameliorating CBF (30).

### Effects of TQ on asthma

Asthma is a common chronic respiratory diseases characterized by airway inflammation and obstruction in the lung (8). Asthma symptoms consist of coughing, wheezing, hardness of the chest and breath shortness (31–33). Inflammatory cells such as eosinophils, mast cells, neutrophils and macrophages play a main role in the pathogenesis of asthma by realizing inflammatory mediators and free radicals. The role of oxidative stress in the pathophysiology of asthma is supported through experimental studies (34, 35). Several investigations indicated that *N. sativa* may be effective for asthma treatment due to its anti-inflammatory and antioxidant properties of the main flavonoids such as TQ (36).

In this context, Al-Majed and colleagues indicated that TQ has a protective effect on the tracheal responsiveness in the guinea-pig. The result showed that the TQ decrease in tracheal responsiveness to carbachol. TQ effect on tracheal responsiveness was significantly enhanced with nordihydroguiaretic acid, quinacrine and also methylene blue. Generally, TQ inhibited the effects of serotonin and histamine on the ileum smooth and tracheal muscles of the guinea-pig. The study suggested that the relaxation effect of TQ may be related to the prevention of lipoxygenase products through blocking of non-elective receptors of serotonin and histamine. The findings of the study confirmed the traditional use of black seeds for asthma treatment (37).

The study conducted by El Gazzar et al. indicated the protective effects of TQ on airway inflammation in allergic asthmatic mice. Induction of allergic airway inflammation was performed by intraperitoneal sensitization and airway challenge through nasal inhalation. Mice (3 males and 3 females/group) were sensitized on day 0 and 14 by intraperitoneal injection of 20 *μg* of OVA. For TQ treatment, mice were injected (intraperitoneal) once daily for 5 days preceding the first OVA challenge, with 3 *mg/kg* TQ in 10% DMSO, or an equal volume of 10% DMSO alone. The results showed that intraperitoneal administration of TQ could reduce the lung eosinophilia and increase the levels of Th _2_ -cytokines in the Bronchoalveolar Lavage Fluid (BALF) of OVA-sensitized mice. Furthermore, TQ reduced the levels of OVA-specific IgE, IgG _1_ , IL-4, IL-5 and IL-13 as well as increased the IFN-γ in the BALF of sensitized animal. The histopathological study indicated that TQ decreased eosiniphilic inflammation and also goblet cells mucus in the lung tissue. The findings of the study suggested that TQ decreased allergic airway inflammation by preventing infiltration of eosinophil and Th2 cytokines in the airways (38).

The smooth muscle relaxant activity of TQ has been reported by Ghayur et al. Guinea-pig tracheal tubes were dissected out and kept in Kreb’s solution, and glucose 11.7; pH 7.4. Tracheal tube was cut into rings, 2–3 *mm* wide, then mounted in a 20 *mL* tissue bath containing Kreb’s solution maintained at 37*°C* and aerated with carbogen gas. A preload tension of 1 *g* was applied to each of the tracheal strips. The tissue was equilibrated for 1 *hr*, after which contractile responses to submaximal concentrations of CCh (1 *μM*), intervals of 45 *min*, were recorded until reproducible responses were obtained. We then tested the effect of the test compound on resting baseline tension of the tracheal strip as well as against CCh (1 *μM*) and high K+-(80 *mM*) induced contractions. To test for an involvement of β-adrenergic receptors in the relaxant effect of the test compound, the tissues were pretreated for 1 *hr* with propranolol (1 *μM*), and response of test compound was repeated in the presence of the antagonist upon CCh-induced contractions. The result indicated that TQ (10, 100, 1000 *μM*) caused non-adrenergic relaxation of agonist-induced contractions in guinea-pig trachea. TQ also blocked the ACh-induced airway narrowing and Ca^++^ signaling in airway smooth muscle cells. The results also confirmed the use of TQ containing plants for treatment of asthma (39).

In the pathophysiology of asthma, Leukorienes (LTs) are known as important inflammatory mediator which increased in reaction to allergen exposure. El Gazzar et al. investigated the effect of TQ on LTs synthesis in OVA-sensitized mice. Lung inflammation was induced by intraperitonial sensitization and airway challenge through nasal inhalation with OVA allergen. Mice (3 males and 3 females/group) were sensitized on days 0 and 14 by i.p. injection of 20 *μg* of OVA. Two weeks after the second sensitization, mice were airway challenged three times (days 28–30) via nasal inhalation with 1% OVA/saline aerosol for 20 *min*. For TQ treatment, mice were (i.p.) injected once daily for the 5 days preceding the first OVA challenge, with 3 *mg/kg* TQ in 10% DMSO or an equal volume of DMSO alone. All mice were analyzed 24 *hr* after the last OVA challenge. Pretreatment with TQ prevented expression of 5-lipoxygenase (the main enzyme in leukotriene synthesis) and also decreased the levels of LTB4 and LTC4 in lung cells. Furthermore, TQ decreased eosinophilia and Th2 cytokines in lung tissue. The study showed the anti-inflammatory property of TQ in asthma (40). Ammar et al. indicated the preventive effects of TQ and Curcumin (CMN) on the biological indices of asthma. Mice (N=40) were sensitized by subcutaneous injections with 25 *μg* of OVA adsorbed on 1 *mg* of alum in 200 *μL* of normal saline per mouse on days 0, 7, 14. Intranasal challenges with OVA (20 *ng/50 μL* saline) were carried out on days 31, 33, 35 and 37. Mice in the treated groups were treated with oral administration of 10 and 15 *mg/kg/day* of TQ and CMN, respectively, starting from day 30 to day 38 (1 *hr* before challenge on days of challenges), while control group receive drug vehicle (0.5% CMC). The results indicated that TQ was more effective to reduce inflammation in lung tissue and BALF that CMN.

Additionally, the suppressive effect of TQ on TGF-β1 and iNOS was more than CMN. The findings of the study proposed that TQ had more effective role against inflammatory alternations in asthmatic animals (41). Another study examined the prophylactic property of TQ on White Blood Cell (WBC) count in lung lavage and tracheal responsiveness in OVA- sensitized guinea pigs. The results showed that treatment with TQ (20 and 40 *μM*) and Fluticasone Propionate (FP) ameliorated WBC count alternations in sensitized animals. The study indicated the inhibitory effect of TQ on tracheal responsiveness and inflammatory cell count in lung lavage was similar or even more than that of FP (42).

El Aziz and co-workers investigated the effect of TQ on airway hypersensitivity in OVA-sensitized rat. Additionally, Rat Peritoneal Mast Cells (RPMCs) were used to study release of histamine from them. Also, to investigate the effect of TQ against hypersensitivity, anaphylactic shock method (systematically) by compound 48/80 was done. The result indicated that pre-treatment with TQ (3 *mg/kg*, i.p for 5 days) reduced the tracheal spirals to histamine and acetylcholine in OVA sensitized animals. TQ (8 *mg/kg*, i.p) ameliorated the levels of lipid peroxidation (LP), glutathione depletion (GSH), IL-1β, and TNF- α and the inflammatory cells infiltration in both lung tissue homogenates and BALF in animals exposed to endotoxin LPS. TQ (8 *mg/kg*, i.p) also decreased the histamine release from RPMCs (43). The study done by Kalemci and colleagues showed that TQ administration (3 *mg/kg*, i.p) reduced the histopathological alternations in the lung of OVA- sensitized mice (44).

Other study evaluated the prophylactic property of TQ (single dose, i.p) on tracheal responsiveness and lung inflammation in OVA-sensitized guinea pig. Pretreatment with TQ (3 *mg/kg*, i.p), reduced tracheal responsiveness to methcholine, histopathological alternations and eosinophilia in the BAL of sensitized animal. The findings of the study indicated the protective effect of TQ for asthma treatment (45).

The effect of TQ on lung inflammation has been studied in OVA-sensitized mice (N=30). The anti- neovascularization properties of TQ against Vascular Endothelial Growth Factor (VEGF) induced Human Umbilical Vein Endothelial Cells (HUVECs) have been studied. Mice were sensitized with 10 *μg* OVA (i.p.) on days 0, 14 and 21 days. After two weeks, mice were exposed to 1% OVA through nebulizer for 30 *min* three times a week for 8 weeks. TQ was given mice from days 15 to 56 an hour before every nebulization (1% OVA. The findings showed that TQ treatment decreased the inflammatory response and neovascularization by prevention VEGF expression. The study suggested that TQ may be useful for asthma treatment by modulating inflammation (46). Pejman et al. investigated the anti-asthmatic mechanism of TQ in the presence of selective A _2A_ and A _2B_ adenosine receptor antagonists in seventy OVA-sensitized guinea pigs. Thymoquinone and each of these antagonists with 3 *mg/kg* dose were injected i.p on 10^th^ day of sensitization protocol. Tracheal Responsiveness (TR) to methacholine and Ovalbumin (OA), and total and differential cell count in Lung Lavage fluid (LLF) in different groups were measured. The result indicated that TQ ameliorated neutrophil, basophil and eosinophil numbers in the LLF and tracheal responsiveness to OVA and methacholine in the sensitized group. Significant reduction in EC50, LLF lymphocyte, monocyte and neutrophil as well as elevation in tracheal reaction to OVA, LLF total WBC and eosinophil number were seen in the sensitized group pretreated with A _2A_ compared to sensitized group pretreated with TQ. Significant increase in the number of monocyte and eosinophil in the LLF in the sensitized group pretreated with antagonists A _2B_ compared to sensitized group pretreated with TQ has been observed. There was a significant increase in the number of eosinophil as well as decrease in the number of monocyte and neutrophil in the LLF of sensitized group pretreated with TQ + antagonists A2A compared with the sensitized group pretreated with TQ. According to the study, some of the anti-inflammatory properties of TQ may be related to the adenosine receptors which are affected by TQ (47).

### Effect of TQ on pulmonary fibrosis

Pulmonary Fibrosis (PF) is a lung disorder that occurs following lung tissue damage. The lungs alveoli undergo stiffness that causes difficulty in breathing and getting insufficient oxygen into the bloodstream. The breathing is worsened during fibrosis, however; medications may improve respiratory function and life quality (48–52). The study evaluated the anti-fibrotic effect of TQ against bleomycin (as anti-cancer chemotherapy medication) which induced oxidative stress and inflammation in the lung of forty eight rats. Administration of TQ (5 *mg/kg*), 1 week before and during bleomycin treatment (5 *mg/kg/day*) decreased lung weight and LDH, WBC, total protein and bronchoalveolar mucin. Additionally, TQ ameliorated the levels of Nitric Oxide (NO) and lipid peroxides, SOD and Slutathione Transferase (GST) activity. Also, TQ improved the alveolar emphysema, inflammatory cell influx, per bronchiolar inflammation via modulating nuclear factor kappa-B (NF-B) expression in lung tissue. Evaluation of hydroxyproline in the group treated with bleomycin was modulated with TQ treatment. Moreover, histopathological findings confirmed anti-fibrotic property of TQ (53). Pourgholamhossein et al. evaluated preventive and therapeutic properties of TQ and its molecular mechanism on lung fibrosis induced by Paraquat (PQ) (as a herbicide) in mice (N=40). Pulmonary fibrosis was induced by i.p. administration of a single dose of 20 *mg/kg* PQ. Pulmonary fibrosis developed in 2 weeks, confirmed by morphological changes in the lungs and excessive accumulation of interstitial collagen. Administration of TQ (40 *mg/kg*, orally), ameliorated PQ induced histopathological alterations. In addition, TQ reduced lipid peroxidation and hydroxyproline content. TQ reversed α-SMA, TGF-β1, collagen 1a1 and collagen 4a1 genes expression to the control level. The findings showed that TQ improved PF through prevention of oxidative stress and profibrotic genes down-regulation (54).

### Effect of TQ on lung cancer

Lung cancer is recognized as a most common cancer in the word. Small Cell Lung Cancer (SCLC) and Non-Small Lung Cancer (NSCLC) (responsible for 15 and 85% cases, respectively), are two main types of lung cancer (55–57). TQ has been indicated to have anti- tumor properties in breast, ovarian and colon cancer derived cells (58–62). In this context, Jafri and co-workers studied TQ effect on lung cancer by using *in vivo* and *in vitro* models. TQ prevented cell proliferation, decreased cell viability and caused apoptosis. TQ (100 *μM*) with cisplatin (5 *μM*) prevented cell proliferation approximately 90% which this combination had a synergistic effect. Furthermore, TQ changed the extracellular condition preventing the invasion and decreasing the ENA-78 and Gro-alpha cytokine production, which have role in neovascularization. In mice xenograft model was found that TQ and cisplatin combination significantly decreased tumor weight and size without further poisoning in mice. The combination of TQ (5 *mg/kg*) and cisplatin (2.5 *mg/kg*) decreased tumor size in a dose dependent-manner. In addition, NF-kappaB expression was down regulated by TQ. The study explained that the combination of TQ and cisplatin may be effective for lung cancer treatment (63).

Ulasli et al. investigated the effects of TQ, resveratrol and Caffeic Acid Phenylester (CAPE) on oxidative stress markers, inflammatory parameters, mRNA expression levels of protein and lung cancer cells surviving by using *in vitro* model. TQ treatment decreased Bcl2 (as an inhibitor of apoptosis) and increased Bax (as pro- apoptotic) proteins in cell. Additionally, TQ and CAPE increased Bax expression. TQ and benzo(a)pyrene plus resveratrol (RES) decreased Bcl-2 expression. However, all three compounds reduced cyclin D expression and elevated p21 expression, but TQ showed the most increase in p21 expression. TQ, RES and CAPE increased expression of TRAIL receptor 1 and 2. TQ and RES decreased IKK1and NF-kappa B (an active player in human cancers) expression. Viability of treated cells with all three agents significantly reduced compared to the control group. The study indicated the anti-tumor activity of TQ, RES and CAPE may be related to increase in their regulatory effect on p53 levels (64). Yang et al. investigated the TQ effects on cellular proliferation, immigration and invasion and also its anti-metastatic mechanisms in lung cancer cell line A549 cells. The findings indicated that TQ could prevent the multiplication, immigration and invasion of A549 cells. In addition, TQ inhibited cyclin D1, PCNA, MMP2, and MMP9 expression at 10, 20, 40 *μmol/L* doses. Furthermore, TQ prevented expression of cell cycle inhibitor P16 and the MMP2 and MMP9 gelatinase functions and decreased phosphorylation of ERK1/2. The study confirmed the therapeutic effects of TQ to treat of human lung cancer (Table 1) (65).

**Table 1.** Provides a brief summary of protective effects of thymoquinon on pulmonary disorders

**Experimental model**	**Effect**	**Ref.**
Rat	Protective effect on lung against ALI and ARDS induced by intratracheal induction of human gastric juice	15
Mice	Decreased Th2 cytokines, lung eosinophilic inflammation and goblet cell hyperplasia. Also it inhibited COX-2 protein expression and PGD2 generation induced by OVA	16
Guinea pigs	Ameliorated pathological changes of lung and reduced OVA induced increased blood IL-4 levels.	17
Mice	Protect mice from DEP-induced reduction of SBP and leukocytosis, elevated IL-6 concentration and diminished plasma SOD activity	18
Rat	Decreased peribronchial inflammation, alveolar edema, alveolar septal infiltration, alveolar exudate, interstitial fibrosis and necrosis following chronic toluene exposure. It also decreased the action of in situ apoptosis recognition by TUNEL, the iNOS and an increase of lung tissue surfactant protein D expression.	19
Rat	Decreased the changes in lung and serum parameters with inflammatory responses and also less antioxidants restoration and fat peroxidation, decreased secretion of serum TNF-α induced by CP	20
Rat	Reduced oxidative stress and histopathological damages following acute abdominal aorta ischemia-reperfusion.	21
Rat	Reduced elevated *LOOH* and total *SH levels increased by* HBO_2_ therapy	22
Rat	Improved mesenteric hypoperfusion and decreased CLP induced aortic impairment. It also inhibited CLP induced elevation of serum ALT, AST, LDH, Cr, BUN, TNF-α, IL-1 β and IL-6. Furthermore, it prevented decrease of glutathione liver, spleen and kidney and increase in MDA level in lung, liver, spleen and kidney.	23
Mice	Prevented tracheal constriction by Ba (2+). It also blocked leukotriene-d induced tracheal contractions. At high concentration it slightly improved the rate of mucociliary transport.	29
Guinea-pig	It reduced tracheal smooth muscle tension, eliminated the increasing effects of serotonin and histamine on the ileum smooth and tracheal muscles	37
Mice *In vitro* (lung cells)	Reduced the lung eosinophilia and increased levels of Th2-cytokines seen following airway stimulation with OVA in the BALF and after challenge of lung cells with OVA, reduced the increased levels of OVA-specific IgE and IgG1., prevented eosiniphilic inflammation in lung tissue and goblet cells mucus-producing induced by allergen, prevented IL-4, IL-5 and IL-13 secretion in the BALF.	38
Mice lung	Prevented Ca(++) signaling in smooth muscle cells of airways and airway restriction induced by Ach	39
Mice	Prevented expression of 5-LPO by lung cells and decreased LTB4 and LTC4 levels, caused to decrease eosinophilia in lung tissue and Th2 cytokines. in allergic asthma induced by OVA	40
Murine model	Suppressed lung inflammation and reduced serum IgE and suppressive effects on TGF-β1 and iNOS related to asthma	41
Guinea pig	Improved total WBC, lymphocytes and eosinophils alternations and tracheal reaction in OVA- induced sensitivity	42
Guinea pig *In vitro* (RPMCs)	In sensitivity induced by OVA, it reduced the reaction of the tracheal spirals to histamine and Ach, inhibited reaction to the endotoxin LPS such as LP, GSH, IL-1β, and TNF- α levels and the inflammatory cells infiltration in both lung tissue homogenates and BALF, prevented release of histamine from RPMCs.	43
Mice	It reduced histopathological alternations of OVA- induced chronic asthma.	44
*In vitro* (well differentiated human sinonasal epithelial cells)	It elevated human CBF.	30
Guinea pig	Reduced tracheal reaction, histopathological alternations and presence of eosinophils in bronchoalveolar lavage in methacholine or OVA- induced asthma Decreased the inflammatory response (antagonizing IL-4/-5 production) in asthma induced by OVA, showed anti- neovascularization effect (prevention from expression of VEGF by VEGFR2/PI3K/Akt signaling pathway).	45
Mice, *In vitro* (HUVECs)	It elevated EC50 and LLF neutrophil number and reduced tracheal reaction to OVA and smethacholine, basophil and eosinophil numbers in LLF.	46
Guinea pig	Decreased enhanced lung weight and LDH, WBC, total protein and bronchoalveolar mucin, restored increased NO and lipid peroxides levels and reduced SOD and GST, neutralized alveolar emphysema, inflammatory cell influx, peribronchiolar inflammation and activated form of NF-B over expression in lung fibrosis induced by bleomycin	47
Rat	Ameliorated PQ induced histopathological alternations, reduced lipid peroxidation and HP content, reversed α-SMA, TGF-β1, collagen 1a1 and collagen 4a1 genes expression.	53
Mice	Ameliorated PQ induced histopathological alternations, reduced lipid peroxidation and HP content, reversed α-SMA, TGF-β1, collagen 1a1 and collagen 4a1 genes expression.	54
*In vitro* (NSCLC, SCLC), Mice	Prevented cell proliferation, decreased cell viability and caused to apoptosis, prevented cell proliferation and changed the extracellular condition preventing invasion and decreasing the ENA-78 and Gro-alpha cytokine production, decreased tumor weight and size without further poisoning for mice, NF-kappa B expression was down regulated by TQ.	63
*In vitro* (lung cancer cells)	Decreased Bcl2 and increased Bax expression, increased the Bax/Bcl2 ratio, reduced cyclin D expression and elevated p21 expression, increased expression of TRAIL receptor 1 and 2 and decreased IKK1 and NF-kappa B expression.	64
*In vitro* (lung cancer cell line A549 cells)	Prevented the multiplication, immigration and invasion of A549 cells, inhibited cyclin D1, PCNA, MMP2, and MMP9 expression, prevented expression of cell cycle inhibitor P16 and the MMP2 and MMP9 gelatinase functions and decreased phosphorylation of ERK1/2.	65

ALI: acute lung injury, ARDS: acute respiratory distress syndrome, Th: T helper cell, COX: cyclooxygenase, PGD2: prostaglandin D2, OVA: ovalbumin, IL: interlukin, DEP: diesel exhaust particle, SBP; systolic blood pressure, SOD: superoxide dismutase, dUTP: deoxyuridine triphosphate, TUNEL: terminal deoxynucleatidyl transferase iNOs: inducible nitric oxide synthase, TNF: tumor necrosis factor, CP: cyclophosphamide, LOOH: lipid hydro-peroxide, SH: total thiol, CLP: cecal ligation and puncture, ALT: alanine transaminase, AST: aspartate aminotransferase, HDL: high-density lipoprotein, Cr: creatinine, BUN: blood urea nitrogen, MDA: malondialdehyde, NDGA: nordihydroguiaretic acid, Qn: quinacrine, HBO2: hyperbaric oxygen, BALF: bronchoalveolar lavage fluid, Ach: acetylcholine, Ig: immunoglobulin, LT: leukotriene, TGF: transforming growth factor, LPS: lipopolysaccharide, GSH: glutathione peroxidase, RPMC: rat peritoneal mast cell, CBF: cilliary beat frequency, VEGF: vasoendothelial growth factor, PI3K:phosphatidylinositol 3-kinase, LLF: lung lining fluid, NO: nitric oxide, SOD: superoxide dismutase, GST: glutathione-S-transferase, NF-B: nuclear factor kappa-B, HP: hydroxyproline, SMA: alpha smooth muscle actin antibody, ENA: epithelial-neutrophil activating peptide, Bax: BCL2 associated X, TRAIL: tumor necrosis factor-related apoptosis-inducing ligand, MMP: matrix metalloproteinases.

## DISCUSSION AND CONCLUSION

Herbal medicine has been considered for the prevention and treatment of various diseases for years. Because of low- cost and availability of medicinal plants, interest to use them is increasing compared to chemical drugs. *N. sativa* (black seed) has been known as a considerable commercial medicinal plant with anti- inflammatory, anti-oxidant, anti-microbial and anti-hypertensive properties (66). The anti-inflammatory and anti-oxidant features of black seed can inhibit and decrease of the problems of neoplasm (67). These features of black seed are related to its main compounds particularly TQ. Several investigations showed anti-inflammatory and anti-oxidant effects of TQ (58, 68). Therefore, our review analyzed the therapeutic effects of TQ against pulmonary disorders related to anti-oxidant and anti-inflammatory effects.

TQ showed anti-inflammatory effect via decreasing the expression of COX-2 and 5-LPO (the main enzyme in leukotriene synthesis), the serum levels of TNF-α, IL-1 β, IL-4, IL-5 IL-6, and IL-13, Th2, LTB4, LTC4 and PGD2. Furthermore, TQ reduced the eosinophil count in the lung tissue. On the other hand, TQ caused trachea relaxation via inhibition of LPO products following metabolism of arachidonic acid and possibly through non-elective receptors blocking of serotonin and histamine. TQ also prevented asthma by modulating oxidative stress. Additionally, TQ was effective against pulmonary fibrosis through prevention of oxidative stress and down-regulation of pro-fibrotic genes. TQ showed anti-tumor activity by inducing apoptosis and decreasing inflammation. Prevention of cellular proliferation, immigration and invasion and also anti-metastatic mechanisms in lung cancer cell were observed by TQ administration. In conclusion, all searched papers confirmed that TQ dose-dependent has therapeutic effect on pulmonary diseases via inducing cytotoxicity and reducing the inflammatory indexes. It is also expected that the present review would be a guidance and motivation for the researchers to investigate preclinical and clinical trials on the use of TQ for treatment of pulmonary disorders.
